# Exploring Non-conventional Dental Implants Beyond Traditional Paradigms Part I: Bridging the Gap in Bone Deficiency Cases

**DOI:** 10.7759/cureus.74271

**Published:** 2024-11-22

**Authors:** Mostafa I Fayad, ِAbdel Naser M Emam, Khaled Mashoor Hyderah, Fatemah B Ibrahem, Omar M Alaryani, Mohammad A Alqhtani, Mohammed H Alyami, Ayidh F Almakhalas

**Affiliations:** 1 Department of Substitutive Dental Sciences, College of Dentistry, Taibah University, Medina, SAU; 2 Department of Prosthetic Dental Science, Faculty of Dentistry, Najran University, Najran, SAU; 3 Department of Prosthodontics, Faculty of Dental Medicine, Al-Azhar University, Cairo, EGY; 4 Department of Preventive Dental Science, Faculty of Dentistry, Najran University, Najran, SAU; 5 Department of Dentistry, Tawq Alseha Medical Center, Najran, SAU

**Keywords:** bone disorders, bone loss, mini implant, oral health management, short dental implant

## Abstract

Dental implants have revolutionized tooth replacement, offering a functional and aesthetically pleasing alternative to traditional dentures and bridges. While conventional implants, typically titanium screws placed into the jawbone, have become the gold standard, many studies explore non-conventional implant designs and materials to address specific challenges and patient needs. This series of literature reviews aimed to delve into non-conventional dental implants, examining their unique features and applications and the current state of evidence supporting their use.

The short and mini dental implants represent a cutting-edge area of research within the field of implant dentistry. Its potential application in the management of cases with limited bone availability has emerged as a viable alternative to the use of bone augmentation procedures.

To date, significant progress has been made in the field of dental implants, particularly with the introduction of short and mini dental implants in the management of cases with significant bone deficiency. However, it remains a remarkable challenge that continues to be actively researched.

## Introduction and background

Conventional dental implants, often referred to as endosteal implants, consist of a titanium screw placed into the jawbone, promoting osseointegration (bone fusion). Non-conventional implants deviate from this standard approach, employing alternative materials, designs, or placement techniques to address specific challenges [1-4]. These challenges can arise from various factors, including the following: The first is insufficient bone. In cases of significant bone deficiency, the conventional implants may not have sufficient bone to anchor to, leading to instability and potential failure. Another is limited surgical accessibility. Due to complex anatomy, some jaw areas, such as the posterior maxilla, may be difficult to access for conventional implant placement. The non-conventional implants aim to overcome these limitations by offering alternatives to the standard titanium screw design and placement methods [5].

Search strategy

**Figure 1:**
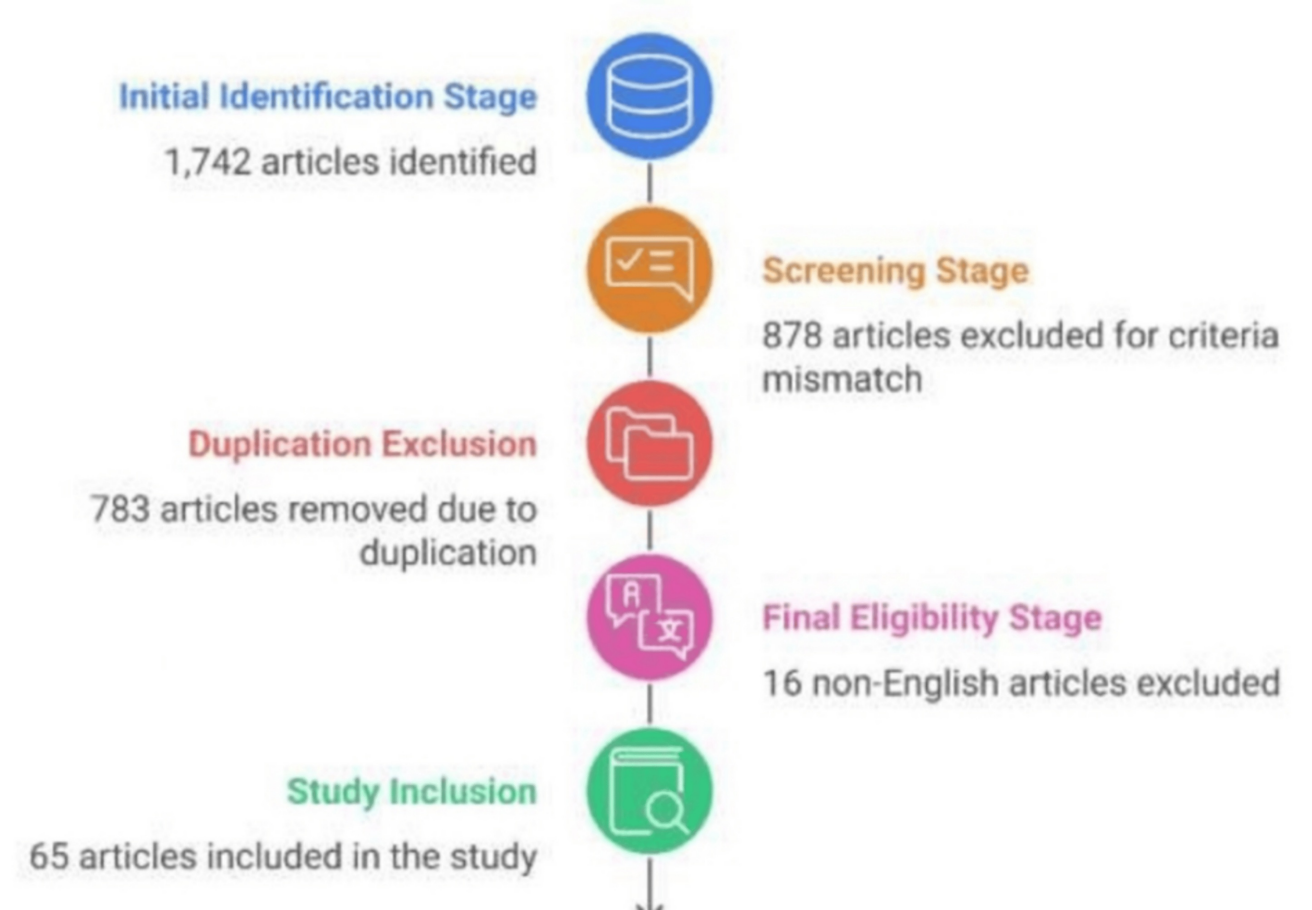
Article selection flowchart

The primary databases searched included PubMed/Medline, Scopus, Web of Science, and Google Scholar for broader coverage. The employed search key terms were "non-conventional dental implants" and specific variations like "short implants" and "mini implants". (AND, OR) were used to combine keywords effectively. Filters were set to limit results to the articles in English published in peer-reviewed journals, including clinical studies, clinical trials, comparative studies, meta-analyses, systematic reviews, reviews, and case reports. Inclusion criteria emphasized studies focusing on non-traditional implants used in cases of insufficient bone and their clinical outcomes, including studies published up to October 2024. Articles on orthodontic mini implants were excluded as these differ significantly in purpose and function from dental implants used for prosthetic support. Case reports with limited relevance were excluded. In the initial identification stage, a total of 1,742 articles were identified through database searches using relevant keywords. During the screening stage, 878 articles were found to be incompatible with the inclusion criteria and were excluded from further consideration. Additionally, 783 articles were excluded due to duplication. In the final eligibility stage, 65 articles were included in the study after excluding 16 non-English articles. This rigorous process ensured that only relevant and high-quality articles were selected for the research. The process of the article selection is summarized in the article selection flowchart (Figure 1). The presented findings are organized under the following subheadings: characteristics of short and mini dental implants (length and design), comparison between short and mini dental implants, loading protocols for short and mini dental implants, factors influencing the choice of loading protocol, factors that influence clinical success, prosthetic material and restoration type, indications for use, clinical scenarios warranting short and mini dental implants, types of prosthetic restorations suitable for short/mini implants, survival rates of short/mini implant versus standard implants, potential adverse effects related to short and mini implants, and maintenance and care for prosthetic restorations.

## Review

Short and mini dental implants in bone deficiency cases

These implants are designed to address the challenges posed by limited bone availability, a prevalent issue in many patients requiring prosthetic rehabilitation. Understanding the characteristics and distinctions between these implant types is crucial for effective treatment planning and achieving optimal outcomes [6,7].

Short and mini dental implants are emerging as viable treatment options for patients facing the challenge of limited bone height and width [8,9]. These implants, characterized by their reduced dimensions, offer a less invasive and more predictable alternative to traditional bone augmentation procedures [10]. This comprehensive review delves into the prosthetic applications of short and mini dental implants in managing limited bone availability, exploring their effectiveness, advantages, and limitations. Using short implants in situations of limited bone height has gained traction due to the inherent challenges of managing atrophic jaws [11]. Bone resorption, a natural process after tooth loss, can reduce the vertical and horizontal dimensions of the alveolar bone. This bone loss can significantly impede the placement of conventional-length implants, often necessitating complex and time-consuming bone grafting procedures [12,13].

Short and mini dental implants offer a less invasive approach to restoring function and aesthetics in patients with limited bone height. These implants are designed to be placed directly into the existing bone, eliminating the need for extensive bone augmentation. This minimally invasive approach reduces the patient's discomfort, surgical time, and the risk of bone grafting complications [14,15].

The short implants are particularly beneficial in the posterior regions of the jaws, where bone height is often limited due to anatomical structures like the inferior alveolar nerve and the maxillary sinus [16,17].

This review aims to comprehensively understand the prosthetic applications of short and mini dental implants in managing limited bone availability. This comprehensive review will provide a valuable resource for dental professionals seeking to understand the applications of short and mini dental implants and their role in managing limited bone height and width.

Characteristics of short and mini dental implants (length and design)

As the name suggests, short dental implants are characterized by their reduced length, typically measuring less than 10 mm. This design is particularly advantageous when conventional implants are not feasible due to insufficient vertical bone height. Short implants are commonly used in the posterior regions of the jaws, particularly the maxilla, where limited bone availability is often a concern. They provide a viable alternative to more invasive bone grafting procedures, minimizing surgical intervention and reducing patient discomfort [14].

The design of short dental implants can vary depending on the manufacturer, but generally, they are characterized by a platform-switched design. This means that the implant body is wider than the implant platform, which helps to improve the stability and biomechanical performance [16]. Short implants can also feature different surface treatments that promote better osseointegration and improve the implant's long-term survival rate. Moreover, short implants often incorporate locking tapers and plateau root shapes, contributing to their stability and resistance to occlusal forces. These design features aim to mitigate the potential disadvantages associated with the reduced surface area of short implants compared to conventional implants [9,18].

Mini dental implants are made in one piece. However, conventional implants usually consist of two parts. Mini dental implants are characterized by their smaller diameter, typically 2.4-3.3 mm. Their reduced size allows for placement in areas with limited bone width, making them suitable for patients with narrow alveolar ridges. Mini dental implants are often used to support overdentures, providing improved retention and stability compared to traditional dentures. They can also be used for single-tooth replacements in specific clinical scenarios [19,20].

The design of mini dental implants typically features a one-piece tapered implant, simplifying the placement procedure and reducing the need for complex surgical techniques. Mini dental implants are often placed flapless, minimizing tissue trauma and accelerating healing. This minimally invasive approach is favorable for patients with limited bone availability or compromised oral health [7].

Comparison between short and mini dental implants

Short and mini dental implants share the common goal of addressing the challenges associated with limited bone availability and providing viable solutions for patients with insufficient bone height or width without requiring extensive bone grafting procedures. However, they differ in their primary characteristics. Short implants are shorter but maintain a standard diameter, while mini implants are smaller in diameter but may have a comparable length to conventional implants. The choice between short and mini implants depends on the clinical scenario and the patient's needs [20,21]. Table 1 presents the comparison of standard, short, and mini dental implants [22-24].

**Table 1:** Comparison of standard, short, and mini dental implants

Feature	Standard implant	Short implants	Mini implants
Length	10 mm or longer	<10 mm	Variable, often comparable to conventional implants
Diameter	Ranging from 3.75 mm to 5 mm	Wider diameter to compensate for length	Smaller, typically 2.4-3.3 mm
Applications	General-purpose, suitable for most cases	Posterior regions, single-tooth replacements	Overdentures, single-tooth replacements in specific cases
Placement	Requires adequate bone height and width	Requires adequate bone width	Suitable for narrow alveolar ridges

Loading protocols for short and mini dental implants

The choice of loading protocol for short dental implants is a critical aspect of treatment planning, as it can significantly impact the success and longevity of the implant. The optimal loading protocol depends on complex factors, including bone quality, implant design, and patient-specific characteristics [25,26].

The results of many studies suggest that both immediate and early loading protocols can be successful for short implants, with high survival rates and minimal bone loss. However, careful patient selection and meticulous treatment planning are essential to ensuring optimal outcomes. Factors such as bone quality, implant design, and prosthetic design can influence the success of these protocols. The evidence suggests that while immediate and early loading protocols can be successful for short implants, further research is needed to refine these protocols and optimize their application in various clinical scenarios [20,27].

A meta-analysis by Kulkarni et al. investigated the clinical outcomes of early and immediate loading protocols for short dental implants (6 mm), including implant survival and marginal bone loss. The meta-analysis reported a pooled implant survival rate of 91.63% and a mean bone loss effect estimate of 0.52 mm. The study found no statistically significant difference in bone loss between immediate and early loading protocols. However, it identified a moderately positive correlation between the crown-to-implant ratio and mean bone loss levels, suggesting that loading protocol may indirectly impact bone loss through this ratio [21].

The choice of loading protocol for mini dental implants, similar to conventional implants, significantly impacts their success and long-term stability. While immediate loading is often employed for mini dental implants, the clinical outcomes and survival rates associated with different loading protocols remain a subject of ongoing research. The radiographic and clinical outcomes of immediately loaded dental implants supporting a mandibular overdenture over three years were evaluated in many studies. The clinical evaluations included plaque index, gingival index, and probing depth values, while radiographic assessments measured vertical and horizontal alveolar bone loss. After three years, the studies reported favorable peri-implant tissue responses of immediately loaded mini dental implants supporting a mandibular overdenture [28,29].

Maryod et al. [30] evaluated and compared the clinical outcomes of immediate and early loaded mini implants supporting mandibular overdentures; they reported that the early and immediate loading protocols showed good clinical outcomes with favorable peri-implant tissue response three years after implant insertion.

The different loading protocols in implant dentistry for partially dentate arches were evaluated to assess the outcomes early. They immediately loaded implants included in comparative, cohort, and retrospective studies as well as prospective case series. Existing evidence suggests that implant survival is not affected by implant placement or loading protocols, provided that careful patient assessment and planning have been undertaken [31].

Factors influencing the choice of loading protocol

The choice of loading protocol should be individualized based on factors such as bone quality, implant characteristics, and patient-related factors.

Bone Density

Often classified using the Lekholm and Zarb classification (D1-D4), it significantly determines the optimal loading strategy. Denser bone types (I-II) exhibit greater strength and resilience, while softer bone types (III-IV) are more prone to resorption and potential implant failure. Denser bone types generally are more amenable to immediate loading, while softer bone types may benefit from a delayed or progressive loading protocol [32,33].

Implant Design

It also plays a crucial role in determining the appropriate loading protocol. Factors such as implant diameter, thread design, and surface topography can influence the stability and biomechanical performance of the implant, impacting the choice of loading protocol. Wider-diameter implants, for example, often exhibit greater stability and are better suited for immediate loading. Conversely, smaller-diameter implants may require a more conservative approach, such as delayed loading, to ensure adequate osseointegration and stability [34].

The tapered implant achieved greater primary stability values measured with insertion torque values (ITVs) and less marginal bone loss than the cylindrical implants. Tapered implants showed a higher insertion torque when compared with cylindrical implants. Therefore, the tapered implants should be used for the immediate loading protocol because of their better primary stability [35].

The implant with a progressive thread design was compared with other implant designs; the studies demonstrated higher bone-to-implant contact percentages immediately after insertion [36].

Modifying the thread profile can influence the contact area between the implant and the bone along the outer surface of the implant threads. An increased bone-to-implant contact area can enhance primary stability. However, if the stresses generated are excessive, it can trigger osteogenic cellular remodeling, leading to bone resorption, reduced implant stability, and potential clinical failure. Thus, any changes to the macro-geometry aimed at improving primary stability should focus on minimizing compressive stress while maintaining normal bone healing patterns, which are crucial for long-term osseointegration [37].

Patient Factors

Age, medical history, overall health, and the patient's level of personal oral hygiene and periodontal status are of fundamental importance for the future success of osseointegration and can also influence the choice of loading protocol. Patients with compromised systemic health conditions may benefit from a delayed loading protocol to allow for sufficient time for healing and osseointegration. Conversely, healthy patients with good bone quality may be suitable candidates for immediate or early loading, potentially achieving faster functional restoration [38,39].

Factors that influence clinical success

The successful use of short and mini implants depends on careful patient selection, considering factors such as the following.

Bone Quality

The density and quality of the bone surrounding the implant site are crucial for osseointegration. While short implants have been shown to be effective in moderate bone quality, careful evaluation is necessary to ensure adequate bone support [40,41].

Bone quality plays a crucial role in implant osseointegration and long-term stability. Studies have shown that short implants placed in D2 bone quality exhibit favorable outcomes, indicating moderate bone density. Calvo-Guirado et al. [40] observed that most patients (88%) in their study presented with D2 bone type, suggesting that short implants can be successfully placed in moderate bone density.

However, short implants placed in the maxilla, where bone density is typically lower, may have higher failure rates. A study by Van Doorne et al. [7] observed a tendency toward higher failure rates for mini implants placed in the maxilla supporting a maxillary overdenture after a five-year follow-up. Similarly, Demiralp et al. [13] found that placed region, age, and bone quality had statistically significant effects on the survival rate of short implants. This highlights the significance of careful patient selection and meticulous treatment planning when considering short implants in patients with limited bone height.

Anatomical Considerations

The proximity of vital structures should be carefully assessed to ensure safe implant placement. The anatomical structures, such as the nasopalatine canal and accessory canals, may influence the implant outcome.

Occlusal Forces

Short implants may be less resistant to occlusal forces due to their smaller surface area. It is essential to carefully plan the prosthesis to distribute occlusal loads effectively and minimize the risk of implant failure [42].

Implant Design

Particularly the surface topography and thread design can significantly impact the biomechanical performance of short implants. Alqahtani et al. [16] investigated the biomechanics of short implants with different diameters and thread designs in D4 bone quality. Their findings demonstrated that wider-diameter implants exhibited lower stress, strain, and micromovement levels, suggesting better stability under load. Square thread designs also yielded the most favorable biomechanical parameters compared to other thread shapes. These findings suggest that optimizing implant design, particularly with wider diameters and square thread designs, can enhance the stability and success of short implants in patients with limited bone height.

Anniwaer et al. [43] evaluated the impact of prosthetic index structures (cross-fit vs. torc-fit) and implant materials (titanium vs. titanium-zirconium) on stress distribution in implant restorations through finite element analysis and digital image correlation. Key findings include that while changing the implant material significantly influenced the maximum stress in the implant itself, it did not notably affect stress in other components. The torc-fit group demonstrated better overall stress distribution and structural integrity than the cross-fit group, suggesting improved reliability in implant systems.

Prosthetic material and restoration type

Sabău et al. [44] investigated the impact of restoration type and prosthetic material on peri-implant bone resorption in a Romanian population, analyzing 160 implants over six years. The key findings indicate that zirconia restorations lead to higher bone resorption than metal-ceramic restorations. Regarding the restoration type, the implant-supported dental bridges had significantly higher resorption than implant-supported crowns.

The choice of prosthetic material for short implants can also influence treatment outcomes. While traditional metal-based prostheses have been widely used, metal-free materials, such as fiber-reinforced composite (FRC) bars, are gaining popularity. These materials offer several advantages, including improved aesthetics, biocompatibility, and reduced cost [45].

Patient Factors

Busenlechner et al. stated that smoking and periodontal conditions double the implant failure rate. It has been seen that when a patient's age increases, the failure rate has a tendency to increase. Multiple studies have highlighted a relationship between advanced age and the incidence of peri-implantitis. Various variables, particularly common in older persons, contribute to this correlation, including difficulties sustaining adequate dental hygiene, reduced masticatory function, and decreased bone mineral density [46-48].

Implant Placement Aftercare

The patients should be informed of potential complications and the need for regular maintenance. After implants are placed in the edentulous area, regular maintenance, follow-up evaluations, and radiographic assessments are necessary to ensure the longevity of the restorations. It is essential for the dental implant team to be proficient in implant maintenance procedures, as implant failure can lead to professional scrutiny and undermine credibility. These maintenance protocols are typically scheduled at specific intervals to help patients maintain optimal oral implant health. The studies highlighted that postoperative care protocols should prioritize regular oral hygiene and follow-up evaluations to effectively monitor and maintain peri-implant tissue health over time [49-51].

Indications for use

The utilization of short and mini dental implants in prosthetic applications for managing limited bone width and height is becoming increasingly prevalent. These implants offer a less invasive alternative to traditional bone augmentation procedures, which can be costly and time-consuming and carry inherent risks. This minimally invasive approach can be particularly helpful for patients who are not suitable candidates for extensive surgical interventions due to medical conditions, anatomical constraints, or financial limitations. The decision to use short or mini implants should be based on a careful assessment of the clinical scenario, patient factors, and specific prosthetic goals [52].

Clinical scenarios warranting short and mini dental implants

The use of short and mini implants can be particularly beneficial in the following clinical scenarios.

Posterior Edentulous Regions

Short implants have shown promising results in restoring posterior edentulous areas, particularly in the mandible [53].

Maxillary Sinus Lift

In situations where sinus elevation is required, short implants can be placed without the need for extensive bone grafting, reducing the complexity and potential complications of the procedure [54,55].

Anterior Maxilla and Atrophic Posterior Mandible

Mini implants are indicated in the edentulous or partially edentulous arch when the facial-lingual width of the bone is inadequate for the placement of a traditional width implant. Mini implants are also used in the anterior maxilla because of decreased palate-labial bone width and/or insufficient interdental space. In the atrophic posterior mandible, where insufficient buccolingual bone width may be found, it is the common indication for mini implant placement [56,57].

Types of prosthetic restorations suitable for short/mini implants

Overdenture

Short and mini implants may be used to support overdentures, providing improved retention and stability for patients with severely resorbed jaws. The overdentures supported by short and mini implants offer significant advantages over conventional dentures, including improved retention, stability, and chewing function [58].

The minimum number of mini implants required to retain complete removable dentures properly may be six in the maxillary arch and four in the mandible. A study comparing mini and short implants in completely edentulous patients with atrophic ridges found that mini implants exhibited a more favorable effect on the supporting structure, suggesting greater long-term stability [59]. However, it is essential to note that the study had a limited sample size and follow-up period and further research is necessary to confirm these findings.

Removable Partial Dentures

Short implants can be used as anchors for removable partial dentures, providing additional support and stability. This approach can benefit patients with multiple missing teeth and limited bone height [60].

Single Tooth Replacements

Short implants can effectively replace missing teeth with single crowns, particularly when bone height is limited. Mini and short dental implants provide a simpler and more predictable treatment option compared to longer implants, minimizing the need for invasive procedures [17,51].

The anterior sites may be more appropriate because of lower occlusal forces and short interdental space (less than 5 mm), such as maxillary lateral and mandibular incisors, and sites where tooth movement has imposed on the site length or the local anatomy is diminutive may accept a single mini implant [19].

Fixed Bridges

Short and mini dental implants can support fixed bridges, especially for shorter spans. However, longer spans may require additional implants or alternative solutions to ensure stability [5,19].

Survival rates of short/mini implant versus standard implants

Several studies have reported favorable survival rates for short and mini implants, demonstrating their efficacy in managing limited bone height. A systematic review by Carosi et al. [27] analyzed 14 full-text publications and found that short implants (6 mm) exhibited survival rates ranging from 92% to 96.9% over a 1-5-year follow-up period in patients with severe mandibular atrophy. Similarly, a retrospective study investigated the cumulative survival rates of Bicon short implants (<8 mm) over a five-year period, reporting a survival rate of 97.3%. These findings suggest that short implants can achieve comparable survival rates to standard-length implants, particularly in cases where bone augmentation is not feasible. 

A retrospective study by Lee et al. [61] evaluated the survival rates of mini implants placed in nine edentulous patients who received four mini implants in the anterior region. The study reported a survival rate of 97.2% after one year, demonstrating the potential for mini implants to provide long-term stability.

A systematic review by Lemos et al. [57] analyzed 24 studies evaluating the use of mini implants to retain complete overdentures in 1273 patients. The review revealed a high overall survival rate of 92.32% for mini implants, with a follow-up time ranging from one to 17 years. The review also highlighted a higher failure rate in the maxillary arch (31.71%) compared to the mandibular arch (4.89%).

A retrospective study by Bungău et al. [62] examined the failure rate of mini implants in 432 patients. The study found that the highest rejection rate occurred within the first month (15.2%), with significantly higher failure rates observed in the buccal mandibular region compared to the palatal region.

Potential adverse effects related to short and mini implants

While short and mini implants offer a viable alternative to traditional implants in managing limited bone available, it is essential to consider potential adverse events associated with their use. One common concern is marginal bone loss, which can affect the implant's long-term stability. A meta-analysis by Kulkarni et al. [26] reported a mean bone loss effect estimate of 0.52 mm after loading for short implants, suggesting that minimal bone loss can occur. However, the study also found a positive correlation between the crown-to-implant ratio and mean bone loss levels, indicating that higher ratios may lead to increased bone loss. This suggests careful attention should be paid to the crown-to-implant ratio when designing prosthetic restorations for short implants.

Another potential complication is implant failure. While studies have generally reported high survival rates for short implants, evidence suggests that certain factors can increase the risk of failure. For instance, Renouard and Nisand [63] reported higher failure rates for short implants placed in the maxilla and sites with bone grafts. The patients older than 60 years had higher failure rates than younger patients. These findings underscore the importance of considering patient-specific factors and implant placement considerations to minimize the risk of implant failure.

This available evidence suggests that these implants can be a reliable option for patients. However, they should be used in conjunction with appropriate prosthetic restorations and careful patient selection.

Maintenance and care for prosthetic restorations

Regular Dental Checkups

Regular dental checkups are essential for monitoring implant health and identifying any potential issues. These checkups should include professional cleaning and radiographic evaluations [49].

Occlusal Adjustments

Occlusal adjustments may be necessary to ensure a proper bite and minimize stress on the implants. Regular occlusal checks can maintain optimal function [18].

Prosthetic Maintenance

Patients should be advised to report any problems or concerns to their dentist promptly [5].

Oral Hygiene

Maintaining excellent oral hygiene is crucial for preventing peri-implant disease. Patients should be instructed on proper brushing and flossing techniques, as well as the use of interdental cleaning aids [64,65].

Future directions for research in short and mini dental implants

While significant progress has been made in understanding the applications of short and mini dental implants, long-term clinical studies are needed to optimize implant designs, including thread patterns, surface modifications, and platform switching, to improve biomechanical performance and reduce stress on the surrounding bone. More research is needed to evaluate the optimal prosthetic designs and loading protocols for short and mini dental implants in various clinical scenarios.

Recommendations for practitioners

Based on the evidence reviewed, the following recommendations can be made for practitioners: First, consider short and mini dental implants as a treatment option for cases with limited bone availability. They are a valid alternative to bone augmentation procedures and can provide comparable outcomes with reduced risk. Second, carefully select implant designs and placement strategies to optimize stability and biomechanics. The success of implants is affected by implant diameter, thread design, and crown-to-implant ratio. Third, implement strict clinical protocols and follow-up procedures to monitor implant performance. Regular assessments are crucial to identify potential complications and ensure long-term success. Lastly, educate patients about the benefits, limitations, and risks of short and mini dental implants. Informed consent is essential to ensure patient satisfaction and manage expectations.

## Conclusions

Short and mini dental implants represent a significant advancement in implant dentistry. These implants offer a less invasive and cost-effective alternative to traditional implants, making them a valuable treatment option for patients with limited bone height. While studies commonly demonstrate high survival rates for short implants, there was evidence highlighting factors that may contribute to an increased risk of failure. More studies are needed to evaluate the optimal prosthetic designs and loading protocols for short and mini dental implants in various clinical scenarios.
